# Infant Milk Formulas: Effect of Storage Conditions on the Stability of Powdered Products towards Autoxidation

**DOI:** 10.3390/foods4030487

**Published:** 2015-09-22

**Authors:** Stefania Cesa, Maria Antonietta Casadei, Felice Cerreto, Patrizia Paolicelli

**Affiliations:** Dipartimento di Chimica e Tecnologie del Farmaco, Università degli Studi di Roma “La Sapienza”, Piazzale Aldo Moro 5, 00185 Rome, Italy; E-Mails: mariaantonietta.casadei@uniroma1.it (M.A.C.); felice.cerreto@uniroma1.it (F.C.); patrizia.paolicelli@uniroma1.it (P.P.)

**Keywords:** infant milk formulas, shelf-life, lipid peroxidation, malondialdehyde, colorimetry

## Abstract

Thirty samples of powdered infant milk formulas containing polyunsaturated fatty acids (PUFAs) have been stored at four different temperatures (20, 28, 40 and 55 °C) and periodically monitored for their malondialdehyde (MDA) content up to one year. MDA levels ranged between 250 and 350 ng/kg in sealed samples with a maximum of 566 ng/kg in samples stored at 28 °C for three weeks after opening of their original packages, previously maintained for ten months at 20 °C. Sample stored at 40° and 55 °C were also submitted to CIE (Commission Internationale de l’Eclairage) colorimetric analysis, since color is the first sensorial property that consumers may evaluate. Overall, the results demonstrated a good stability of PUFA-enriched infant milk formulas in terms of MDA content. However, some care has to be paid when these products are not promptly consumed and stored for a long time after first opening.

## 1. Introduction

Infant milk formulas represent the only food for non-breast-fed infants and premature babies, playing a very important nutritional role, since they represent the only way for providing newborns with all of the nutrients they need for healthy growth. Due to the complex nature of infant milk formulas and the stability of some of their components, great care has to be taken in all steps of their formulation, handling and storage [1].

According to the Italian and international laws [2] and GMP requirements, powdered milk formulas are obtained by deep modification of cow milk, with the final aim of simulating breast milk as much as possible [3,4,5]. In particular, they are supplemented during the manufacturing process with polyunsaturated fatty acids (PUFAs), such as linoleic (LA 18:2n-6), α-linolenic (ALA, 18:3n-3), arachidonic (ARA, 20:4n-6) and docosahexaenoic acid (DHA, 22:6n-3) [6], as they are considered important for the proper neural and cognitive development of newborns, in general, and premature babies, specifically [7,8]. However, due to their low chemical stability, the addition of PUFAs to foodstuffs may represent a problem for their shelf life and, consequently, for consumers’ safety, especially when they are designed for infants. Newborns are highly exposed to the toxic effects of the degradation products of PUFAs [9], because of the immaturity of their enzymatic systems and detoxification organs, their low body weight [10] and, above all, because no other options are available for them, other than infant milk formulas.

The main stability problem of all fat-/oil-containing foods is related to the autoxidation process, which is more pronounced when the lipid fraction is sizable, and it gets worse if the composition is changed toward a more unsaturated mixture. Fatty acids with three or more unsaturations represent a class of molecules that can rapidly evolve into unpleasant and toxic by-products [11]. Deterioration of lipid-rich foods starts with the initial formation of peroxides, unstable and reactive primary products, which breakdown into a mixture of off-flavored and potentially toxic, mutagen and carcinogenic [12] secondary products (mainly aldehydes and ketones) with the consequent loss of the nutritional value of the food. For all of these reasons, it is of primary importance to manufacture infant formulas using mild technological processes, low temperature, appropriate packaging and a modified nitrogen atmosphere, in order to protect these products from oxidative decomposition. In fact, infant milk formulas are particularly prone to autoxidation, because of their chemical composition, as they are made of 33% fats and are very rich in polyunsaturated fatty acids [3]. Therefore, the high temperatures required by some steps of the manufacturing process, such as spray-drying, may catalyze the autoxidation process. For these reasons, it is very important to monitor the modification of some oxidation parameters during the storage of milk powder under different conditions, in order to get more insight into their shelf life [13,14]. Among the different parameters that could be used for monitoring the autoxidation process [15,16], to our knowledge, one of the preferred is based on malondialdehyde (MDA) detection [17,18,19], because it allows evaluating the oxidation status of foods in the initial phase of autoxidation. MDA is, in fact, the most important secondary product of autoxidation, and it is usually used as an indicator of the lipid peroxidation process, both for its early appearance and the high sensitivity of the analytical methods available [15]. On this basis, the aim of this study was the evaluation of the stability of infant milk formulas, supplemented with PUFAs, under different storage conditions. The stability of infant milk formulas was evaluated trough the detection of MDA with the thiobarbituric acid (TBA) test, which is the simplest and fastest method available [18,19]. The samples were extracted with aqueous trichloroacetic acid, and MDA was quantified on the basis of the third derivative absorption spectrum of the pink MDA/TBA adduct. Powder milk formulas, sealed in their original packaging, were stored at different temperatures in order to accelerate their eventual oxidation and to have information about the shelf life of these products more quickly. The stability after opening of the products was also monitored simulating the conditions of their domestic use, in order to assess the real exposure risk of newborns to oxidation and other degradation products.

## 2. Experimental Section

### 2.1. Reagents and Samples

All reagents and solvents were of analytical grade. Trichloroacetic acid (TCA), thiobarbituric acid (TBA), butylated hydroxyl toluene (BHT) and 1,1,3,3-tetramethoxypropane (TMP) were purchased from Sigma-Aldrich (Deisenhofen, Germany).

All of the tested infant milk formulas were provided by the international company Kraft-Heinz. They were enriched with LC-PUFAs, Fe and vitamins E, C and A [490 µg retinol equivalents (RE)/100 g powder]. They were received just after manufacturing, sealed in an aluminum cardboard-coated box under a nitrogen modified atmosphere and kept, as received, in a dry place until the analysis. Some of the samples were monitored for three weeks after opening, maintaining them in their original packaging, as in their ordinary use.

### 2.2. Detection of MDA

MDA standard solutions, sample preparation, as well as recovery experiments were performed as previously reported [18].

Briefly, MDA was detected by the TBA test, where one molecule of MDA reacts with two molecules of TBA with the formation of a pink pigment, which can be quantified on the basis of the third derivative of its absorption spectrum.

Stock solutions of MDA were obtained stirring TMP (70 mg) dissolved in 0.1 N HCl (10 mL), for 5 min at 100 °C. Working solutions of MDA were prepared by opportune dilution of the stock solution with distilled water and used for calibration and recovery experiments.

The calibration curve was obtained by plotting the peak height at 526 nm *vs*. MDA concentration in the range 100–2000 ng·g^−1^. It had a correlation coefficient *R*^2^ = 0.9997 and was expressed by: y = 9 × 10^−6^x + 1.0 × 10^−5^, where y is the peak height in V and x is the MDA concentration in ng·g^−1^. The detection limit was 25 ng·g^−1^, and the quantization limit was 80 ng·g^−1^.

Aliquots of MDA stock solution were used also for the recovery experiments. Infant milk formulas were spiked with different amounts of MDA and the recovery yields were calculated as the ratio [(ppb found in the spiked sample)—(initial ppb found in the sample)]/(added ppb), according to Fenaille *et al.* [20]*.* A mean recovery yield of 70% was obtained.

For the quantification of MDA in infant milk formulas, samples (2.0 g) of milk powder were mixed in a test tube with 5% aqueous TCA (8 mL) and 0.8% BHT in *n-*hexane (5 mL). The mixture was stirred at room temperature for 10 min, and then, it was centrifuged. After separation, the top hexane phase was discarded, whereas the cloudy aqueous phase was heated for 20 min at 70 °C and further centrifuged. The aqueous layer was filtered and diluted to a final volume of 25 mL. Aliquots (1 mL) of the obtained solution were mixed with 0.8% TBA in *n*-hexane (1.5 mL) and 5% aqueous TCA (1.5 mL). After incubation carried out for 30 min at 70 °C in screw-capped bottles, the solutions were analyzed by third derivative spectrophotometry carried out with a UV spectrophotometer lambda 40 (Perkin Elmer, Milan, Italy), setting a scan speed of 120 nm·min^−1^ with 1-cm absorption cells. All of the experiments were performed in quadruplicate by two different operators.

### 2.3. Colorimetric Analysis

Infant milk formulas were monitored for color modification. Samples of the milk powder were withdrawn from their packages, placed on Petri dishes and the color measured with an X-Rite SP62 colorimeter, equipped with an integration sphere to determine the color reflectance. The L* (lightness), a* (red/green value) and b* (yellow/blue value) values are related to the illuminant D65 at 10 degrees. Each reported value is the median of ten measurements performed randomly on the surface of the Petri dish. All of the experiments were performed in duplicate.

### 2.4. Statistical Analysis

Each measurement was repeated at least four times, and the results were expressed as the mean ± the relative standard deviations (%RSD). Statistical significance was determined using the GraphPad Prism 4 statistical software package (GraphPad, San Diego, CA, USA). Comparison between experimental data was evaluated by Student’s *t*-test, and statistical significance was defined as *p* < 0.05.

### 2.5. Experimental Planning

All of the samples were stored in a dry place sealed in their original boxes and maintained at three different temperatures of 20, 40 and 55 ± 2 °C, which were regularly monitored throughout the study. At the same time, some of the samples were analyzed for MDA content after opening, simulating the conditions of the ordinary use of milk. To this end, they were stored in their original packages for three weeks at two different temperatures (20 and 28 °C) and analyzed weekly. 

The experimental planning is reported in Table 1.

Samples 1–3 were analyzed for MDA content, according to the above-described procedures, immediately after opening.

Sample 2 was stored for three weeks at 20 °C after opening and analyzed weekly.

Sample 3 was stored for three weeks at 28 °C after opening and analyzed weekly.

Samples 4–7 were stored sealed at 55 °C and opened just before the analysis, which was performed twice a week up to 14 days.

Samples 8–19 were stored sealed at 40 °C and opened just before the analysis, which was performed weekly up to three months.

Samples 20–30 were stored for one year at 20 °C and opened just before the analysis, which was performed monthly.

Sample 25 was stored sealed for six months at 20 °C, then it was opened and kept in its original packaging for three weeks at 20 °C and analyzed weekly.

Sample 29 was stored sealed for ten months at 20 °C, then it was opened and kept in its original packaging for three weeks at 28 °C and analyzed weekly.

Samples stored at 40 and 55 °C were also submitted to colorimetric analysis.

**Table 1:** Experimental planning reporting the storage conditions of the different samples of infant milk formulas.

Sample	Before Opening			After Opening		
	Storage Conditions	Storage Time	Storage Temperature (°C)	Storage Conditions	Storage Time	Storage Temperature (°C)
1	Sample analyzed immediately after manufacturing			-----		
2	Sample analyzed immediately after manufacturing			open	3 weeks	20
3	Sample analyzed immediately after manufacturing			open	3 weeks	28
4	sealed	3 days	55	-----		
5	sealed	7 days	55	-----		
6	sealed	10 days	55	-----		
7	sealed	14 days	55	-----		
8	sealed	1 week	40	-----		
9	sealed	2 weeks	40	-----		
10	sealed	3 weeks	40	-----		
11	sealed	4 weeks	40	-----		
12	sealed	5 weeks	40	-----		
13	sealed	6 weeks	40	-----		
14	sealed	7 weeks	40	-----		
15	sealed	8 weeks	40	-----		
16	sealed	9 weeks	40	-----		
17	sealed	10 weeks	40	-----		
18	sealed	11 weeks	40	-----		
19	sealed	12 weeks	40	-----		
20	sealed	1 months	20	-----		
21	sealed	2 months	20	-----		
22	sealed	3 months	20	-----		
23	sealed	4 months	20	-----		
24	sealed	5 months	20	-----		
25	sealed	6 months	20	open	3 weeks	20
26	sealed	7 months	20	-----		
27	sealed	8 months	20	-----		
28	sealed	9 months	20	-----		
29	sealed	10 months	20	open	3 weeks	28
30	sealed	12 months	20	-----		

## 3. Results and Discussion

Thirty samples of just manufactured powdered infant milk formulas, all derived from the same batch, were stored at different temperatures and analyzed for their malondialdehyde content and colorimetric modifications.

According to the Italian and European laws, the analyzed formulas were prepared following the principles of good manufacturing practice (GMP) using powder demineralized whey milk, vegetable oils, powder skim milk, milk proteins, maltodextrins, lactose, minerals and vitamins. All of the samples were also fortified with microencapsulated LC-PUFAs (arachidonic and docosahexaenoic acids) derived from algal and fish oils.

All of the samples were kept in a dry place sealed in their original boxes and stored at three different temperatures of 20, 40 and 55 ± 2 °C, which were regularly monitored throughout the study. At the same time, some of the samples were analyzed for MDA content after opening and storage in their original packaging for three weeks at 20 and 28 °C, simulating the conditions of the domestic use of milk. Three weeks is a reasonable period for consumption and also the maximum recommended by manufacturers after opening, whereas 20 and 28 °C were chosen considering seasonal in-house temperature variations.

A set of preliminary experiments was run with the aim to determine the data reproducibility: three samples (labeled 1–3) were opened immediately after production and analyzed in order to evaluate the intra- and inter-sample variability. Recovery experiments were also carried out on the same specimens [19]. Seventy percent of MDA was recovered in these experiments; therefore, all of the following results were corrected taking into account this value. Each sample was analyzed in quadruplicate by two different operators.

Mean values and relative standard deviations (RSD) were calculated for each sample separately and for the three samples together: for the first one (226, 243, 289, 210), a mean value of 242 ng was obtained with an RSD of 14%; for the second one (286, 244, 266, 221), 254 ng with an RSD of 11%; for the third one (289, 271, 213, 197), 242 ng with an RSD of 18%. Considering all twelve data together, a mean value of 246 ± 33 ng of MDA per g of sample, with an RSD of 13%, was obtained. This mean error was always confirmed in all of the following experiments.

These preliminary results show how important homogenization before sampling is; in fact, when infant milk formulas are properly homogenized, no differences among specimens from the same batch are obtained.

**Figure 1:**
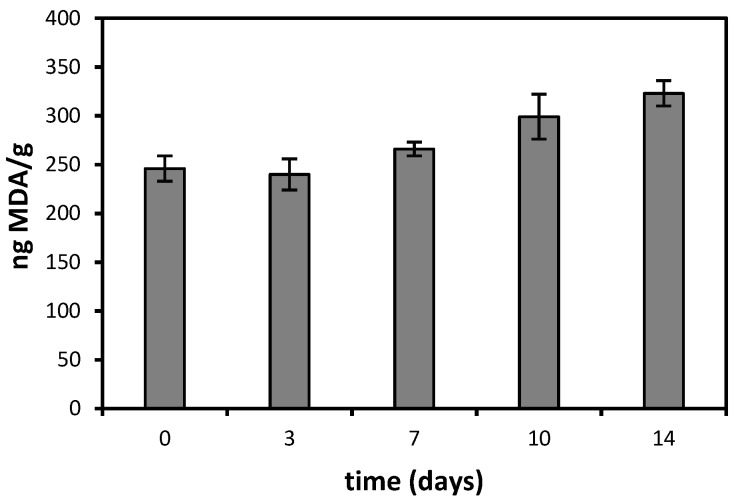
Nanograms of malondialdehyde (MDA) found in 1 g of infant milk formula stored at 55 °C sealed in the original package. Error bars are relative standard deviation (%RSD).

Infant milk formulas were stored at 55 °C (Samples 4–7) and monitored for MDA content during two weeks; on the other hand, Samples 8–19 were stored at 40 °C and monitored weekly up to three months. The data obtained from the experiments carried out at 55 °C are reported in Figure 1. As already described, each result is the mean of four values, previously corrected considering a recovery yield of 70%.

Results from samples stored at 55 °C for three days (240 ng/g MDA) perfectly overlap those obtained from the variability studies; then, a rapid and linear increase of the MDA content can be observed (Figure 1).

The samples were always stored in their original packages, which were sealed during the manufacturing process under a modified atmosphere. Under these conditions, MDA significantly increased by 30% (from approximately 240–320 ng, *p* < 0.05) within 14 days of storage at 55 ± 2 °C, with a linear 10% increment for each data point after the first three days. It is likely that this value is the reduced induction time at 55 °C.

On the contrary, when the samples were stored at 40 ± 2 °C and monitored once a week up to three months, the MDA level varied around a mean value of 255 ng/g, with a mean RSD of 10%, in perfect agreement with the values found in the initial variability experiments.

These results evidenced no significant variation of the MDA content of milk formulas stored at 40 °C for three months, thus indicating a good stability of the analyzed products. At the same time, longer exposition times may be needed under these conditions, in order to have useful information about the shelf life of infant milk formulas, which can be more easily achieved storing the same samples at 55 °C.

**Figure 2:**
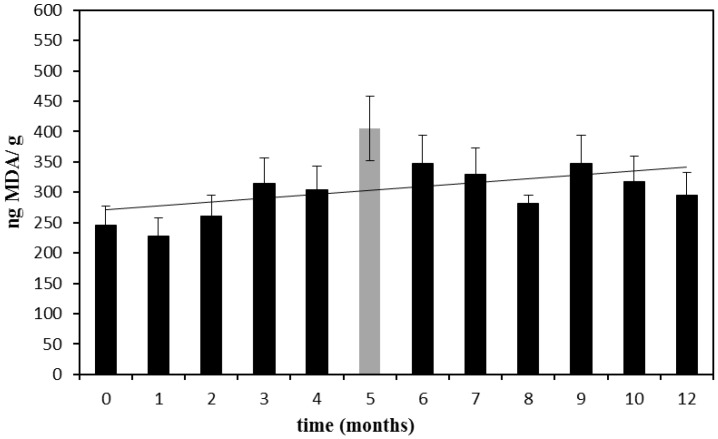
Nanograms of MDA found in 1 g of infant milk formula stored at 20 °C sealed in the original package. The trend-line was calculated excluding the outlier point at the fifth month. Error bars are the %RSD.

Samples 20–30 were stored up to one year at the constantly-monitored temperature of 20 ± 2 °C, and every month, one of the samples was opened and analyzed. The results are reported in Figure 2.

These data showed a weak, but constant increase of MDA, and following the same trend, an increment of about 40% in a year can be assumed. Therefore, a slight increase of MDA levels from a mean value of approximately 240 at time zero (t_0_) to 340 ng/g after one year of storage at 20 ± 2 °C can be supposed.

In the experiments performed at both 20 and 40 °C, MDA levels seem to vary around a mean value with a fluctuating trend that could be explained by the complexity of the matrix [21], and the slight increase becomes evident only after a very long period of storage. This means that 40 °C is too low of a temperature for performing accelerated stability tests and also that the analyzed milks are quite stable towards oxidation.

A set of experiments was also carried out with the aim of simulating the domestic use of infant milk formulas. To this end, Samples 2 and 3 were opened and stored in their original packaging at 20 and 28 ± 2 °C, respectively, and analyzed once a week up to three weeks. These values were chosen considering seasonal variations of in-home temperatures, since they can be considered the normal room temperature and the maximum temperature of a non-conditioned room in summer. At the same time, Sample 25 was stored in its original hermetically-sealed package for six months at 20 °C, and then, it was opened and stored at the same temperature for three weeks. On the other hand, Sample 29 was stored closed for ten months at 20 °C, then it was opened and stored at 28 °C for three weeks.

**Figure 3:**
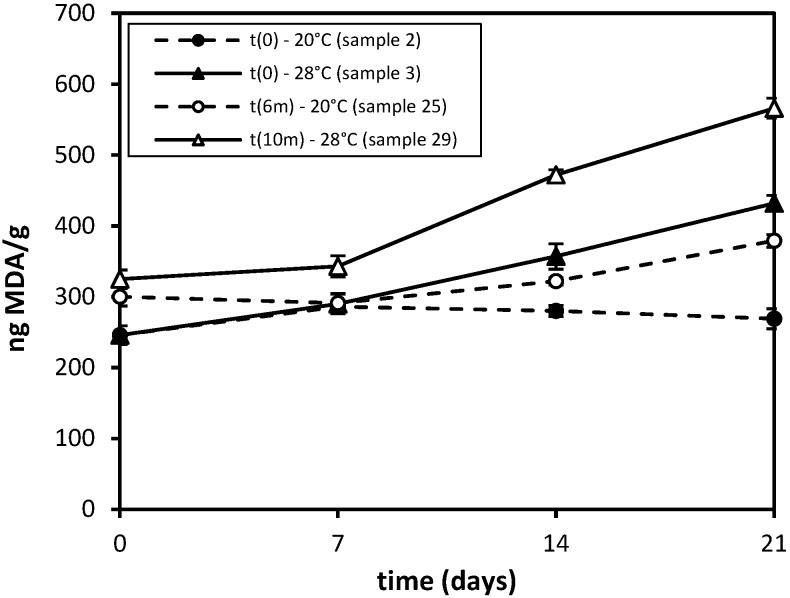
Nanograms of MDA found in 1 g of infant milk formula stored for three weeks, after opening, at 20 or 28 °C. Samples 2 and 3 were analyzed just after manufacturing, whereas Samples 25 and 29 were opened after 6 or 10 months of storage at 20 °C, respectively.

The results of these experiments are reported in Figure 3.

Initial MDA levels for Sample 25 at t_6 months_ and 29 at t_10 months_ are reported as the mean values estimated from the trend line of the data reported in Figure 2.

As is well shown in Figure 3, the MDA levels of Sample 2 fall in the range of the variability study, even if a small increase of 10% can be evidenced with respect to the mean value. On the contrary, Sample 25, analyzed in the same conditions of Sample 2, but after six months of storage, is stable in the first week; then, it shows a more marked increase of 25% of the MDA content (*p* < 0.05). Sample 3 shows a rapid increase of MDA, which raised weekly by 20% till a final value of 432 ng (75% of the initial value, *p* < 0.05). Sample 29, analyzed under the same conditions of Sample 3, but after 10 months of storage sealed in its original package, is almost stable over the first week; then, its MDA content increases by 75%, behaving exactly like Sample 3 (*p* < 0.05).

As a whole, these experiments show that great care should be taken during the storage of infant milk formulas, with particular attention to temperature, but also that significant changes can occur during their use, especially if they are maintained at unsuitable temperatures. In fact, the combination of prolonged storage times (10 months; infant milk formulas expire after two years from manufacturing) at 20 °C and the use over a period of three weeks at 28 °C leads to very high levels (over 550 ng/g) of malondialdehyde per gram of milk powder.

Comparing data obtained from samples stored under different conditions, some correlations can be obtained between samples stored closed in their original packaging at 20 °C and 55 °C and those stored closed at 20 °C and opened at 28 °C. In fact, an exposition time of one or two months at 20 °C is almost comparable to three or seven days at 55 °C, in the same way that a hermetically-sealed milk sample stored for seven months at 20 °C shows the same MDA content of a sample kept open for one week at 28 °C. The projection of these results evidences the dependence of autoxidation from temperature and air exposition, and assuming a linear increase of MDA concentration, one may expect to find the following values: after 30 days, 250 ng/g of MDA in a sealed sample stored at 20 °C, 450 ng/g in a sealed sample stored at 55 °C, 550 ng/g in a sample stored at 28 °C after opening and 700 ng/g in an open sample stored at 28 °C after keeping it for ten months at 20 °C sealed in its original packaging. The obtained results clearly show how 55 °C could be considered a valid temperature for accelerated stability tests, and they also give an idea of the significant changes that milk powder formulas undergo after opening, especially if they are stored after opening at unsuitable temperatures, and, therefore, how mandatory their consumption in a very short time is.

**Figure 4:**
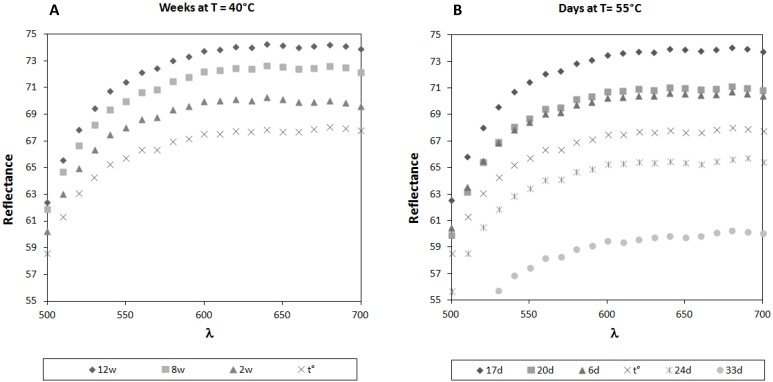
Colorimetric analysis: reflectance spectra of infant milk formulas stored up to 12 weeks at 40 °C (**A**) or up to 33 days at 55 °C (**B**).

To complete and support the data obtained from the MDA analysis, the colorimetric changes suffered by milk powder formulas stored at 40 and 55 °C were evaluated. Interesting results were obtained from the preliminary colorimetric data reported in Figure 4.

Both samples maintained at 40 °C and 55 °C in their original sealed packages show the same behavior: they undergo an initial bleaching, corresponding to a significant increase of both L* (Table 2) and of the reflectance values (*p* < 0.05).

**Table 2:** Effect of temperature on the CIE (Commission Internationale de l’Eclairage) parameters of milk powder formulas.

Storage Temperature (°C)	Storage Time	L*	a*	b*
----	0	84.35	−3.26	15.95
40	2 weeks	85.49	−2.97	15.77
40	8 weeks	86.46	−2.81	16.01
40	12 weeks	86.47	−2.91	17.30
55	6 days	85.65	−3.56	17.23
55	17 days	87.05	−3.63	18.41
55	20 days	85.69	−3.77	18.81
55	24 days	83.03	−3.47	17.51
55	33 days	80.24	−3.04	17.14

L*: lightness; a*: red/green value; b*: yellow/blue value.

This change could be attributed to the bleaching of carotenoid pigments, which were added to the analyzed samples in the form of retinol equivalents (490 µg per 100 g of powder). Similar results have been already reported in the literature [22,23]. In particular, colorimetric analysis of milk powder was previously carried out by Nielsen *et al.* [23] showing a behavior similar to that observed in this work, even if the initial increasing of the L* value was not considered and discussed in depth.

The profile obtained after 17 days at 55 °C corresponds to that recorded after 12 weeks at 40 °C. In fact, the reflectance curve of the samples stored for 17 days at 55 °C completely overlaps that obtained after 12 weeks at 40 °C. After the initial bleaching, the samples maintained at 55 °C undergo a browning process, so that after 20 days of storage, the colorimetric parameters correspond to those obtained at Day 6. After 33 days, the weak decreases of both a* and b* are backed by a significant decrease of L* and of the resulting reflectance (*p* < 0.05). On the other hand, experiments performed at 40 °C for 12 weeks did not show any browning process after the initial bleaching, maybe because the exposition time at this temperature was not long enough for inducing this color change and longer periods of time are required in order to get more complete and useful information. Anyway, although interesting, the obtained results are not sufficient to state whether this temperature is able to start the browning process observed at 55 °C.

This is the first time, to our knowledge, that two different steps in the colorimetric analysis of milk powder stored at different temperatures were taken into consideration: in fact, no bleaching of pigments was discussed before [23,24,25]. The slight modification observed is probably due to the bleaching of carotenoids (the first sign of the beginning of an oxidation process) and not to the Maillard reaction, like browning is. Therefore, this observation could be used to provide information about the shelf life of milk powder formulas in a very short time, even if more investigations are needed to support these results.

As in the case of malondialdehyde detection, also for the colorimetric results, it seems that 40 °C is not a suitable temperature for the prevision of the shelf-life of milk powder formulas, whereas more complete information can be obtained working at 55 °C. The exposition time of 12 weeks at 40 °C was too short to know if the bleaching process was stopped and the time requested to have information appear too long to be compatible with accelerated tests of stability.

To our knowledge, this modification due to the carotenoids’ bleaching, was never evidenced before, as only the browning process due to the Maillard reaction is reported.

## 4. Conclusions

Stability studies performed on just-manufactured LC-PUFAs-enriched infant milk formulas evidenced the good stability of these products towards autoxidation when stored for three months at 40 °C. On the contrary, a significant increase in the MDA content was measured in samples stored for two weeks at 55 °C. Similar MDA levels were obtained after one year of storage at 20 °C. In the same way, preliminary studies on colorimetric changes of milk powder evidenced an initial bleaching of milk followed by a browning process in samples stored at 55 °C for one month, whereas only the bleaching was observed after storage at 40 °C for three months.

Overall, these results suggest that 55 °C may be a suitable temperature to have insight into the stability of infant milk powder formulas, and it might be proposed to predict the shelf life of these products, which have an expiration time of two years, as established by European laws.

In addition, stability studies performed on infant milk formulas after opening of their original packaging evidenced that not only the storage conditions of the sealed products are important, but also the time and temperature after opening have to be considered. In fact, even if low levels of MDA were found in sealed samples stored for a long time at 20 °C, dangerous levels were detected in samples stored open for three weeks at 28 °C. These findings should be used to stress the importance of the storage conditions of LC-PUFA-enriched infant milk formulas, especially in their domestic use.
